# Six-year-old girl with decreased vision

**DOI:** 10.4103/0974-620X.64239

**Published:** 2010

**Authors:** Agha Shahab Haider, Ahmed Al-Hinai, Anuradha Ganesh

**Affiliations:** Department of Ophthalmology, Sultan Qaboos University Hospital, Muscat, Oman

A six-year-old Omani girl presented with complaints of decreased vision in both eyes (OU) of two years duration. Ophthalmic examination revealed best corrected visual acuity of 0.6 OU. Cover test and stereo acuity were normal. Anterior segment examination showed posterior subcapsular cataract OU [Figure 1]. Fundus evaluation revealed a floating mass in the anterior vitreous just behind the lens in the right eye (OD) as shown in [Figure 1]. There was no sign of intraocular inflammation. The fundus examination OU was otherwise unremarkable. B-scan OD showed a opacity in anterior vitreous. Pre-natal history was insignificant. Past ocular history was negative for trauma, infectious or inflammatory ocular disease. The child was otherwise healthy. Routine blood investigations (complete blood picture, erythrocyte sedimentation rate, C-reactive protein) were normal.

**Figure 1:**
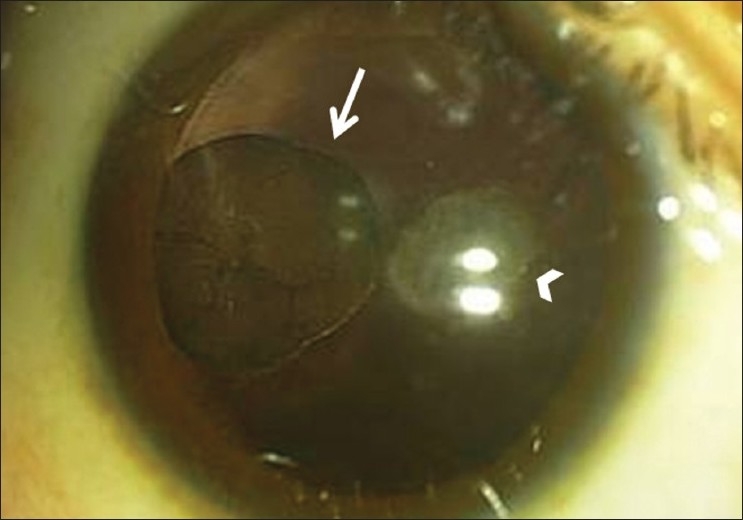
Anterior segment photograph OD showing the posterior subcapsular cataract (arrow-head) and the lesion in the anterior vitreous (straight arrow)

## Questions

1. What is the differential diagnosis for the lesion in the anterior vitreous OD?
2. What is the most likely diagnosis for the above?

## Answers

1. Congenital vitreous cyst, intraocular hydatid cyst, cysticercosis, snowball opacity, Intraocular tumor.
2. Pigmented congenital vitreous cyst.

## Comments

Congenital or acquired cyst of the vitreous is rare. A vitreous cyst may be categorized as occurring in one of the three situations. It may occur in eyes with remnants of hyaloid vascular system, in normal eyes, and in eyes with ocular disease. Acquired cysts have been reported to be usually traumatic or parasitic in origin. There was no history of ocular trauma or surgery in our patient. Parasitic cysts (Echinococcosis and cysticercosis) are often associated with some signs of vitreo-retinal disease and intraocular inflammation. Both these features were absent in our patient. Routine blood tests did not reveal eosinophilia and our patient was otherwise healthy. These features led us to diagnose the lesion in our patient as a pigmented, congenital vitreous cyst.

Pigmented, congenital cysts are believed to originate from the *pars ciliaris* epithelium, while the nonpigmented variety, from remnants of the hyaloidal vascular system. The cyst in our patient was pigmented; more-over we did not find any remnant of the hyaloid system (ie. Cloquet’s canal, glial tissue on the disc). The cyst in our patient therefore could have originated from the pars ciliaris. Congenital cysts may be spherical, oval, or lobulated and its surface may appear smooth or crenellated. They cysts are translucent or semitransparent with an optically clear cavity, and are usually free floating in the midvitreous.

They may also be found in the retrolental space, as in our patient, or more posteriorly near the optic disc. Acquired cysts on the other hand are opaque or barely translucent.

A scolex (head of the echinococcus) might be detected inside the cyst during slit-lamp and/or ultrasound examination in some instances. This was not seen in our patient.

Patients with vitreous cysts occasionally report symptoms of floaters, visual field defects, or intermittent blurring of vision. Argon laser photocoagulation and Nd:YAG lasers have been employed to rupture these cysts. Symptomatic cysts can be aspirated by pars plana approach or can be removed by pars plana vitrectomy.

However as a rule congenital cysts are stable and harmless, rarely interfere with visual acuity, and usually do not require treatment. With respect to our patient, the parents have been informed about the diagnosis and periodic observation without any intervention has been advised

